# Transoral management of adult benign laryngeal stenosis

**DOI:** 10.1007/s00405-020-06210-5

**Published:** 2020-07-23

**Authors:** Fabiola Incandela, Francesco Missale, Francesco Mora, Filippo Marchi, Ivana Fiz, Cesare Piazza, Giorgio Peretti

**Affiliations:** 1 Department of Otorhinolaryngology, Maxillofacial and Thyroid Surgery, Fondazione IRCCS, National Cancer Institute of Milan, Milan, Italy; 2 IRCCS Ospedale Policlinico San Martino, Genoa, Italy; 3 Department of Molecular and Translational Medicine, University of Brescia, Brescia, Italy; 4 Department of Surgical Sciences and Integrated Diagnostics (DISC), University of Genoa, IRCCS Ospedale Policlinico San Martino, Genoa, Italy; 5 Department of Plastic Surgery, Chang Gung Memorial Hospital, Chang Gung University and Medical College, Taoyuan, Taiwan; 6 Department of Otorhinolaryngology, G. Gaslini Children’s Hospital, Genoa, Italy; 7 Department of Oncology and Oncohematology, University of Milan, Milan, Italy

**Keywords:** Stenosis, Larynx, Endoscopy, CO_2_ laser, Outcome

## Abstract

**Purpose:**

Management of benign laryngeal stenosis (BLS) remains challenging even though transoral treatments in selected cases have shown satisfactory results, at least comparable to open-neck approaches, with reduced invasiveness. To date, no overall consensus has been reached on many issues. The aim of this study is to assess the effectiveness of a purely transoral treatment in a cohort of patients affected by BLS.

**Methods:**

We evaluated 40 patients affected by BLS, treated by transoral surgery between 2013 and 2017. The European Laryngological Society classification for laryngotracheal stenosis was applied for the staging. Improvement in airway patency and quality of life was assessed by decannulation rate, Airway-Dyspnea-Voice-Swallowing (ADVS) score, Voice handicap index (VHI)-30, and Eating assessment tool (EAT)-10 questionnaires.

**Results:**

Mean age was 61 years and M:F ratio was 1.4:1. Previous laryngeal surgery was the most common cause of stenosis (50%), followed by radiotherapy (20%), idiopathic etiology (12%), granulomatosis with polyangiitis (10%), and prolonged intubation (8%). Transoral treatment entailed an improvement in quality of life with a significant decrease in the VHI score (*p* < 0.0001) and improvement in Airway (*p* = 0.008), Dyspnea (p < 0.0001), and Voice (*p* < 0.0001) scores. No major perioperative complications were observed. The decannulation rate among patients with a tracheostomy in place (*N* = 16) was 63%.

**Conclusions:**

Transoral treatment of selected BLS managed by a team with high-level expertise in surgery and anesthesiology is associated with significant improvement of quality of life, especially with regard to voice and breathing functions.

**Electronic supplementary material:**

The online version of this article (10.1007/s00405-020-06210-5) contains supplementary material, which is available to authorized users.

## Introduction

Benign laryngeal stenosis (BLS) usually refers to an abnormal narrowing of the upper airway involving one or more laryngeal sites among the supraglottis, glottis, and subglottis. It may be related to various disease processes including iatrogenic causes [post-intubation, post-surgical or post-radiotherapy (post-RT)], inflammatory diseases (granulomatosis with polyangiitis, sarcoidosis, amyloidosis or idiopathic subglottic stenosis), or trauma. Caudal extension of the stenosis to the cervical trachea requires even more complex treatment, which may result in significant morbidity. Furthermore, a small but significant mortality rate ranging between 0 and 8.4% must be taken into account [1, 2].

Management of BLS still represents a surgical challenge even though in the last decades transoral approaches performed in referral centers and in selected cases have achieved results comparable to those previously reported using more traditional open-neck procedures, though with less invasiveness [3, 4].

However, each BLS case may present unique characteristics and should be assessed according to the patient's profile, site, severity and etiology of stenosis. As a result, comparing success rates among different treatment modalities becomes quite difficult since they are not only dependent on the severity of the initial condition, but also on the preoperative evaluation and the ensuing choice of treatment [5, 6].

Further on, the measurement of success in this field was traditionally represented by survival and decannulation rate. However, the introduction of transoral microsurgery for BLS has offered to patients less invasive therapeutic procedures with satisfactory results both in the terms of airway patency and quality of life outcomes [7, 8]. This led to definition of new end points to adequately assess the impact of selected treatment on airway, voice and deglutition function [9–12].

While the ultimate outcome must clearly be to restore and maintain an unobstructed and preferably prosthesis-free airway capable of supporting the patient's ventilatory needs to avoid dyspnea, we must also keep in mind both the preservation of good vocal and swallowing functions as essential therapeutic targets. Accordingly, overall quality of life measurement is becoming increasingly acknowledged as an essential outcome tool in the BLS management arena [10].

The aim of the present study is therefore to assess the effectiveness of exclusive transoral treatment of adult BLS, with the help of quality of life assessment tools, in terms of evaluation of voice, swallowing and airway patency.

## Materials and methods

An observational retrospective study was carried out, enrolling between January, 2013 and December, 2017, 40 patients affected by BLS who were treated solely by a transoral approach at the Department of Otorhinolaryngology – Head and Neck Surgery of the University of Genoa, Italy. Twenty-three (58%) of them were males and 17 (43%) were females (male to female ratio, 1.4 to 1), with a mean age of 60 years (range 14–85). Patients affected by BLS extending to the cervical trachea, or those previously treated by open-neck procedures, or affected by bilateral vocal fold palsy alone were excluded from the present study. Twelve (30%) patients had previously been treated elsewhere by transoral approaches, and 16 (40%) were already tracheostomized at the first consultation in our Department (Table 1).

**Table 1:** Absolute and relative (%) frequencies of categorical variables

Patient characteristics	*N* (%)
All	40 (100)
Gender	
Male	23 (58)
Female	17 (43)
Age	
Mean (range)	60 (14–85)
Etiology	
Post-surgery	20 (50)
Post-RT	8 (20)
Post-intubation	3 (8)
Idiopathic	5 (13)
Granulomatosis with polyangiitis	4 (10)
Previous endoscopic treatment(s)	
No	28 (70)
Yes	12 (30)
Airway or general comorbidities	
No	29 (73)
Yes	11 (28)
Involved sites	
Supraglottis	14 (35)
Glottis	31 (78)
Subglottis	10 (25)
More than one site	15 (37)
Main surgical treatments	
TLM CO_2_ radial incision	10 (25)
TLM CO_2_ posterior cordotomy	8 (20)
TLM CO_2_ lysis of synechiae	20 (50)
TLM cold instrumentation	2 (5)
Adjuvant treatments	
Dilatation	6 (15)
Topical mitomycin-C	10 (25)
Steroid injection	6 (15)
Stent placement	
No	27 (68)
Keel	7 (18)
T-tube	6 (15)
Previous tracheostomy	
No	24 (60)
Yes	16 (40)
Decannulation	
No	6 (37)
Yes	10 (63)

All patients underwent a routine diagnostic work-up including awake flexible videolaryngostroboscopy (Kay Pentax Laryngeal Strobe 9400; Pentax Medical, Montvale, NJ, USA) combined with panendoscopy under general anesthesia by 0° and 70° rigid telescopes using a high-definition television (Olympus Medical System Corporation, Tokyo, Japan). The grade of stenosis was assessed according to the European Laryngological Society (ELS) classification (Table 2) [13].

**Table 2:** ELS BLS classification schema

Grade of stenosis		Sites involved		Comorbidities	
I	≤ 50%	a	One	**–**	No
II	51–70%	b	Two	** + **	Yes
III	71–99%	c	Three		
IV	100%	d	Four		

The etiology of the stenosis was iatrogenic in 31 (78%) patients as it was caused by surgical procedures in 20 (50%), post-RT in 8 (20%), and post-intubation in 3 (8%). BLS was correlated to granulomatosis with polyangiitis in 4 (10%) patients and defined as idiopathic in 5 (12%).

Among the 20 post-surgical stenosis, 16 received previous TLM (15 cases) or open partial laryngectomies (1 case) for laryngeal carcinoma, while 4 cases received TLM elsewhere for bilateral vocal fold palsy evolving in glottic stenosis.

**Fig. 1:**
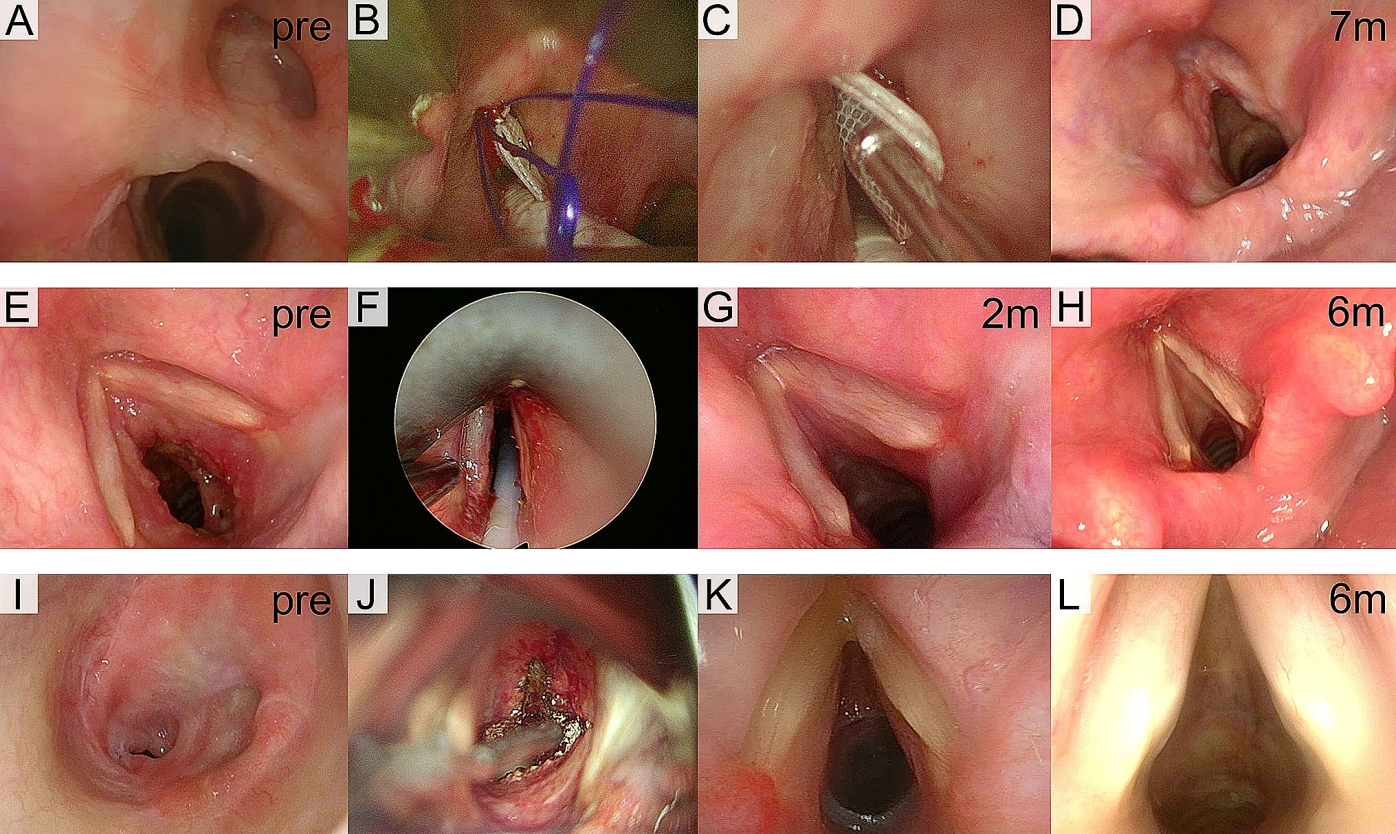
Endoscopic pre-treatment, intraoperative or post-treatment picture of three clinical cases. **a–d** Post-surgical anterior glottic–supraglottic stenosis (ELS IIb) managed by TLM with CO_2_ laser scar excision and stented with a customized Keel removed after 50 days; **e–h** granulomatosis with polyangiitis-associated subglottic stenosis (ELS IIa) treated by TLM with CO_2_ laser radial incisions, steroid infiltration and dilatation, aided by intraoperative use of high-frequency jet ventilation (**f**); **i–l** idiopathic subglottic stenosis (ELS IIIa) treated with TLM CO_2_ laser radial incisions, steroid infiltration, dilatation and stented with a T-tube, achieving the decannulation after 5 months. pre, pre-treatment endoscopic picture; m, months after treatment

Representative endoscopic photo documentation is reported in Fig. 1. According to the ELS classification, 10 (25%) patients were affected by grade I, 9 (23%) by grade II, and 21 (52%) by grade III stenosis. Among them, the stenosis involved one laryngeal site (a) in 25 (63%) patients and two sites (b) in 15 (37%). Eleven (27%) patients were affected by airway or severe systemic comorbidities (sign +) (Table.3).

**Table 3:** Stage of stenosis according to the ELS classification [13]. ELS score obtained the sum of the overall grade of stenosis (I, II or III) with the number of sites involved (*a* = 1, b = 2) and with a further point for relevant comorbidities (+ = 1)

ELS stage	ELS score	*N*	%
Ia	2	7	17.5
Ib	3	2	5.0
Ib +	4	1	2.5
IIa	3	2	5.0
IIa +	4	1	2.5
IIb	4	5	12.5
IIb +	5	1	2.5
IIIa	4	10	25.0
IIIa +	5	5	12.5
IIIb	5	3	7.5
IIIb +	6	3	7.5

### Treatment modality

Surgical treatment included transoral CO_2_ laser microsurgery (TLM) in terms of radial incisions in 10 (25%) patients, posterior cordotomy with partial arytenoidectomy in 8 (20%), lysis of cicatricial synechiae in 20 (50%), with or without local microflap harvesting in 7 and 13 patients, respectively. TLM was always performed by means of a CO_2_ laser (Lumenis Encore Ultrapulse, Tel Aviv, Israel) coupled with an Acublade micromanipulator set at 1–3 W of power, delivered in an ultrapulse modality and continuous mode. In 6 (15%) patients affected by subglottic stenosis, radial incisions were associated with dilatation by means of Savary dilators. Two (5%) patients affected by anterior glottic web were treated by cold knife.

A stent was placed at the end of the surgical procedure in 13 (33%) patients to guarantee a stable airway and to avoid further synechiae formation: a customized silastic Keel stent was placed at the level of the anterior commissure for 8 weeks in 7 (18%) patients who had anterior glottic stenosis, while a Montgomery T-tube was used and left in place for at least 6 months in the other 6 (15%) subjects with subglottic involvement.

Infraglottic high-frequency jet ventilation (HFJV_IG_) was adopted to perform surgical procedures in 10 (25%) patients, thus avoiding tracheostomy in 5 of them, while achieving adequate target exposure that is essential for TLM when dealing with the posterior glottic compartment or with narrow stenosis that is not manageable by standard endotracheal intubation [14].

Medical adjuvant therapy at the end of surgery, in terms of the topical application of Mitomycin-C (at a concentration of 2 mg/ml and kept in place for 2–4 min) and local corticosteroid injections [1–2 ml of Kenacort (triamcinolone acetonide) at a concentration of 40 mg/ml], was administered to 10 (25%) and 6 (15%) patients, respectively.

### Outcome evaluation and statistical analysis

All patients had been evaluated and staged both preoperatively and postoperatively, at least after 6 months or at their last follow-up available (follow-up was updated until May 2018) by the Airway-Dysphonia-Voice-Swallowing (ADVS) staging system [9, 10], the Voice Handicap Index-30 (VHI-30) [12, 15], and the Eating Assessment Tool-10 (EAT-10) [11, 16] questionnaires.

We considered as primary outcome the postoperative quality of life as tested by ADVS, VHI-30, and EAT-10 questionnaires. As secondary outcome we considered the decannulation rate during follow-up for patients with a pre-existing tracheostomy (*N* = 16, 40%) at the time of the endoscopic surgical procedure.

Clinical data are reported with absolute and relative frequencies. Statistical analysis was performed by Chi-square, Fisher's exact or Wilcoxon tests for paired samples, as appropriate.

The effect of the timing variable (pre-treatment, post-treatment) for the quality of life results was investigated with a two-way repeated ordinal regressions and ANOVA type II Sums of Squares analysis [17], testing the interaction with possible confounders: the etiology (post-surgical etiology vs others), previous treatments (no vs. yes), grade of stenosis (I, II or III), number of sites involved (*a* = 1, *b* = 2), presence of comorbidities, and pre-treatment ELS score (the sum of the overall grade of stenosis [I, II or III] with the number of sites involved (*a* = 1, *b* = 2) and with a further point for relevant comorbidities [+ = 1], as shown in Table 3).

In all analyses, a two-tailed *p* value < 0.05 was considered significant. GraphPad Prism (San Diego, CA, USA) and R (version 3.6.2) were used for statistical analysis.

## Results

### Follow-up and events

Median follow-up was 20 months (range 6–60; IQR 14–40); the full time-to-events chart is reported in Supplementary Fig. 1. During follow-up, 8 (20%) patients required a second TLM procedure and 1 (2%) needed a third to obtain a patent and stable airway.

### Questionnaires results

**Fig. 2:**
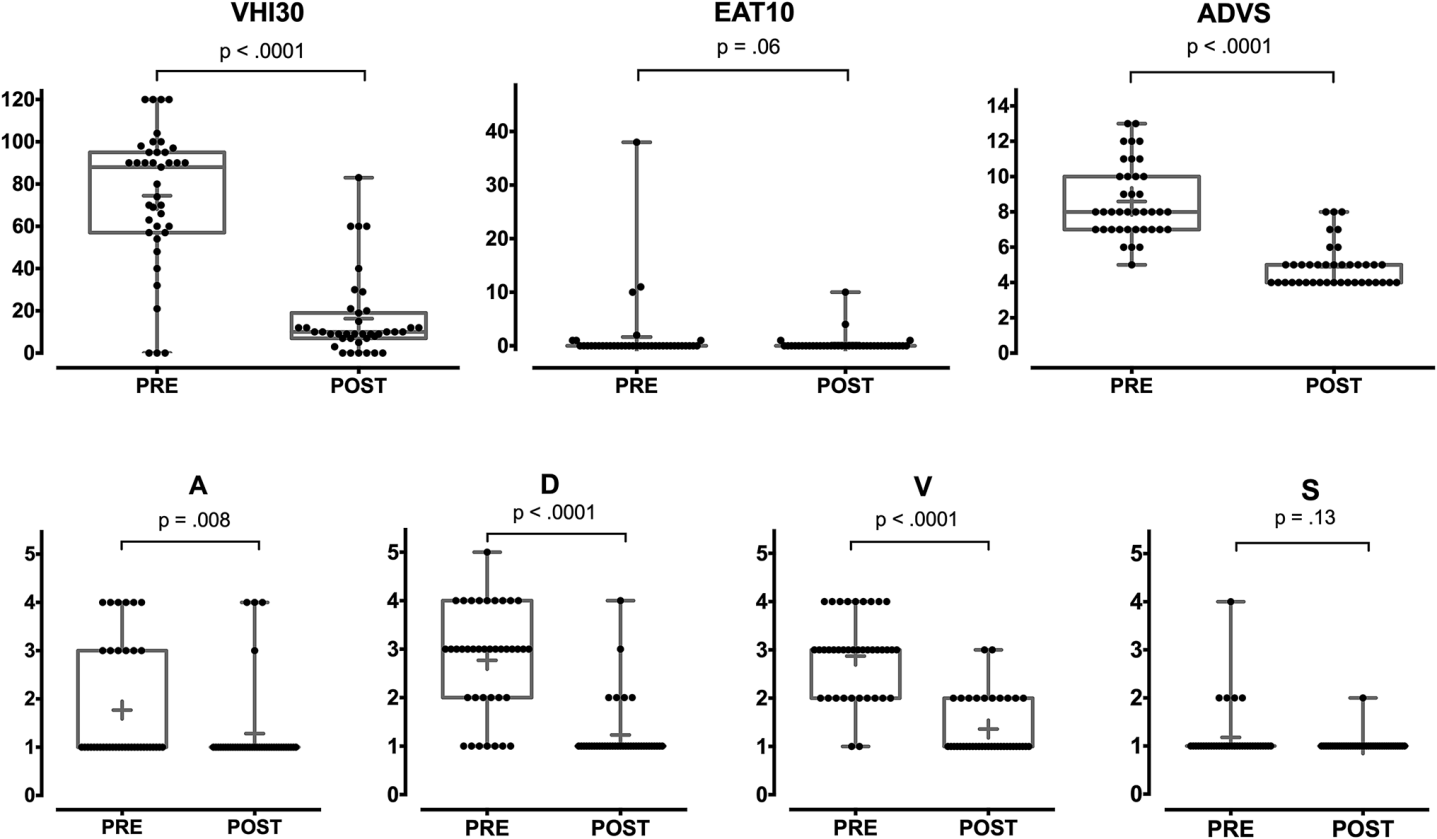
Box plots of VHI-30, EAT-10 and ADVS questionnaire results (*p* values estimated by Wilcoxon test)

The comparison of the quality of life data gathered preoperatively and at the last follow-up visit showed that the VHI-30 score significantly improved [median VHI_pre_ 88 (IQR 59–95) vs. median VHI_post_ 10 (IQR 7–17); *p* < 0.0001]. The functional outcome measured by the ADVS score showed an overall significant improvement: median ADVS_pre_ 8 (IQR 7–10) vs. median ADVS_post_ 4.5 (IQR 4–5), *p* < 0.0001. Analyzing each items of the ADVS questionnaire, the Airway status had also significantly improved [median A_pre_ 1 (IQR 1–3) vs. median A_post_ 1 (IQR 1–1); *p *= 0.008], as had Dyspnea [median D_pre_ 3 (IQR 2–3.5) vs. median D_post_ 1 (IQR 1–1); *p* < 0.0001], and the degree of Voice impairment [median V_pre_ 3 (IQR 2–3) vs. median V_post_ 1 (IQR 1–2), *p* < 0.0001]. On the other hand, swallowing did not show any significant changes, and was within normal range both in the pre- as well as in the postoperative settings [median S_pre_ 1 (IQR 1–1) vs. median S_post_ 1 (IQR 1–1); *p* = 0.13] (Fig. 2).

Self-evaluation of dysphagia by the EAT-10 questionnaire confirmed no changes in swallowing capabilities with normal values in the whole interquartile range [median EAT-10_pre_ 0 (IQR 0–0) vs. median EAT-10_post_ 0 (IQR 0–0); *p* = 0.06] (Fig. 2).

### Quality of life predictors

**Fig. 3:**
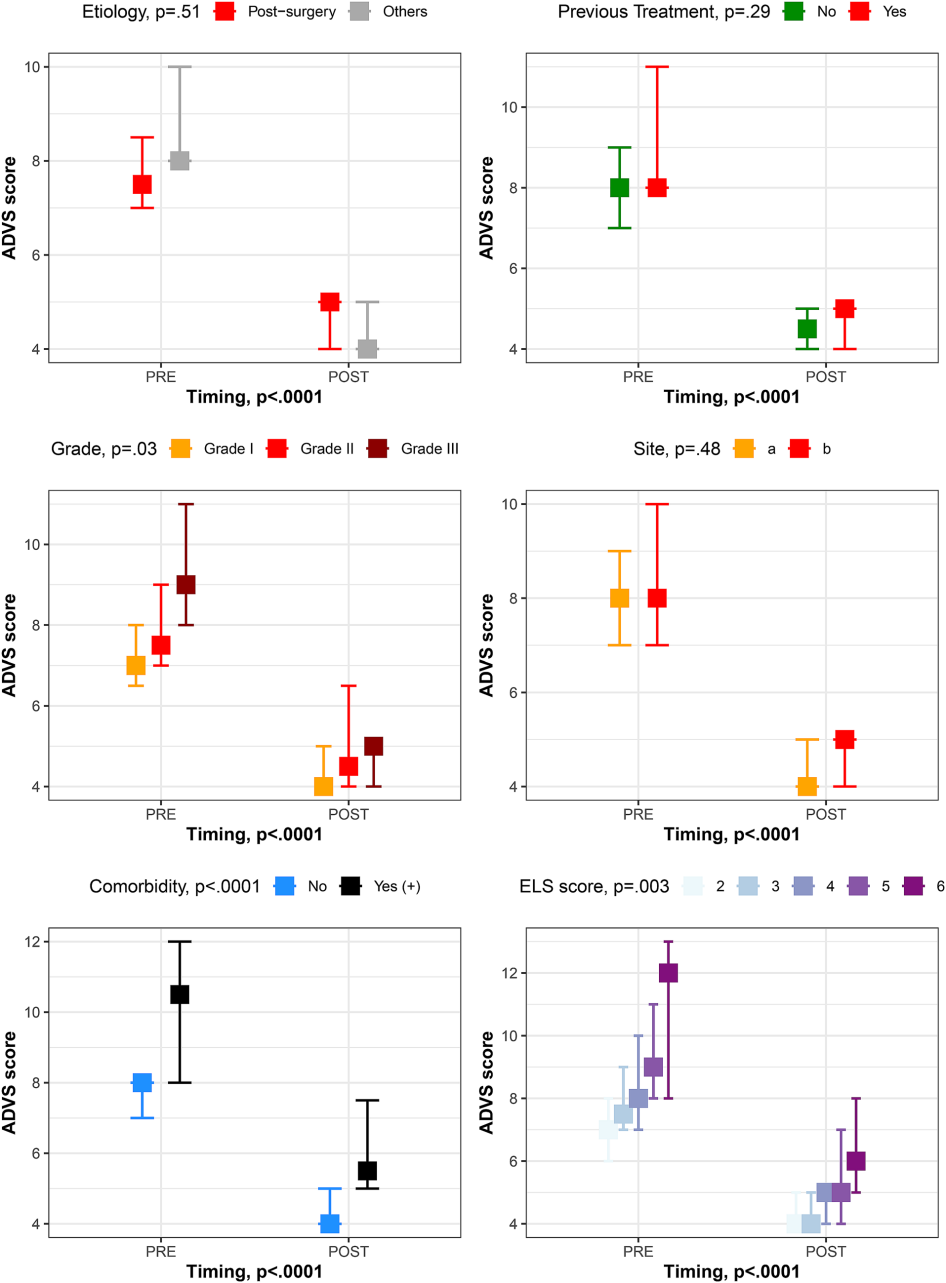
Plots showing pre-treatment (PRE) and post-treatment (POST) median values with 95% CI of ADVS scores in different subgroups of patients according to etiology, previous treatments, stenosis grade, sites involved, presence of comorbidities and pre-treatment ELS score. The main effect of each variable was tested by ordinal regression models, weighed by the timing effect (PRE vs POST); *P* values estimated by ANOVA type II Sums of Squares analysis

The questionnaires results were analyzed by two-way ordinal regression models testing the effect of timing (pre- vs post-treatment results) together with covariates potentially confounders of the results: etiology, previous treatments, number of sites involved, presence of comorbidities and overall ELS score. ADVS scores’ results were ruled out to be associated with different etiology (*p* = 0.51), previous treatments (*p* = 0.29) or number of sites involved (*p* = 0.48), as shown in Fig. 3.

A higher grade of stenosis (*p* = 0.03), presence of comorbidities (*p* < 0.0001) and higher overall ELS score (*p* = 0.003) were associated with a worse ADVS score, obtaining anyway an improvement over time (*p* < 0.0001) (Fig. 3). None of the covariates analyzed were associated with different VHI30 or EAT10 scores (*p* ≥ 0.05).

### Decannulation rate

The presence of comorbidities (sign +) was significantly associated with the presence of, or the need for, tracheostomy (100% vs 17%, p < 0.001). Among the 16 (40%) patients who underwent tracheotomy before or after TLM for BLS, 10 (63%) were successfully decannulated, 2 (12%) preferred to maintain a plugged cannula in situ even though a patent and stable airway had been achieved, 2 (12%) still had a T-tube in place at their last visit, and 2 (12%) remained tracheostomy tube dependent (Table 4).

**Table 4:** Stage and decannulation status at the last follow-up visit of patients with a tracheostomy (*N* = 16)

ELS stage	Decannulation		Tot
	No	Yes	
Ib +	0	1	1
IIa +	1	0	1
IIb +	0	1	1
IIIa	0	5	5
IIIa +	2	3	5
IIIb +	3	0	3
Tot	6	10	16

## Discussion

Based on the recommendations recently put forth by the scientific community, transoral management in our series was strictly limited to carefully selected patients affected by mild–moderate (grade I–III) BLS involving no more than two laryngeal sites [5, 18, 19].

In such a specific clinical setting, a cautious treatment policy was followed due to the negative influence that relevant comorbidities and autoimmune/idiopathic stenosis etiology could have on the final outcome [20–22], being these features reported respectively for 29 (73%) and 9 (23%) patients in the present cohort. In fragile subjects like these, aggressive upfront treatments such as laryngotracheal reconstruction or extended cricotracheal resection and anastomosis are less appealing because of the high complication rates potentially impacting on the postoperative course and quality of life [23, 24]. Moreover, during the diagnostic and therapeutic course, a limited number of transoral and minimally invasive treatments do not preclude further, more aggressive open-neck surgery (if and when needed).

However, to date, there is no general agreement on the ideal therapeutic algorithm to be followed for BLS management, and in fact, treatment tailored to each single patient's characteristics is most often the best therapeutic approach. For example, in case of anterior web-like glottic stenosis, Monnier reported transoral lysis of the synechia with or without harvesting of local rotation microflaps and placement of a silastic keel stent in more advanced stages to be the most appropriate treatment [18]. Other authors recommend transoral surgery followed by adjuvant medical treatments, such as topical application of Mitomycin-C, which is considered useful for minimizing scar formation and preventing subsequent restenosis [25, 26]. When dealing with stenosis limited to the glottic plane, we recommend transoral placement of a silastic keel following TLM for lysis of the anterior commissural web, while in our experience the use of Mitomycin-C was only recommended in case of continuity of the raw mucosal surfaces without full exposure of the cartilage.

With regard to posterior glottic stenosis (PGS), Bogdasarian [27] classified these lesions into four grades ranging from a simple inter-arytenoid mucosal bridge to bilateral arytenoid fixation by a posterior thick scar band, and recommended transoral treatment only for grade I and II stenosis, combined with the open-neck inter-arytenoid posterior augmentation by means of a cartilaginous graft in more severe cases [28, 29]. More recently, other authors confirmed the need to place a cartilaginous graft, thus broadening the possibilities of applying the transoral approach even to cases of PGS with bilateral arytenoid fixation [30]. On the other hand, we successfully treated 8 subjects with bilateral arytenoid fixation caused by a thick inter-arytenoid scarring using transoral unilateral cordotomy [31] combined with medial arytenoidectomy [32], with no graft interposition.

Involvement of the subglottis in BLS represents one of the most crucial issues to be managed due to the anatomical and clinical features promoting the development of stenosis such as reduced blood flow, incomplete support by a damaged cartilaginous framework, exposure to gastroesophageal reflux and turbulent air flow [33]. In such critical cases, and especially in those with concentric subglottic narrowing, we combined TLM radial incisions with delicate dilatations performed by inserting Savary probes to restore acceptable airway patency [34]. The choice of rigid dilators instead of ballooning and/or angioplasty catheters is linked to the surgeon’s choice, giving immediate tactile feedback that allows to modulate the effort needed to dilate the stenosis [35]. A more favorable cost-effectiveness ratio and the immediate availability of such devices are other advantages that cannot be overemphasized. In selected, previously tracheostomized patients in whom PGS extended to the subglottis, a precisely measured Montgomery T-Tube prosthesis tailored to the patient’s subglottic and tracheal anatomy was added after radial incisions and serial dilatations had been carried out. The T-Tube was always well tolerated thus achieving an ultimate success rate comparable to the 71% estimate reported in the literature [36].

In cases of autoimmune and/or idiopathic etiology, adjuvant medical treatment in terms of topical steroid injections or even systemic immunosuppressive therapy [37–39] is strongly recommended after surgical treatment and/or mechanical dilatations. In a recent Polish study, such a therapeutic triad resolved the stenosis after only one procedure in 68.9% of patients [40]. However, it is generally acknowledged that repeat (more than 3), unsuccessful transoral treatments may definitely compromise the ultimate success rate. In light of this, it is of paramount importance to underline that 12 (30%) of the patients in our cohort came to our attention after unsuccessful previous endoscopic treatments, and being 16 (40%) already tracheostomy dependent.

Regarding the functional outcomes reported in the literature, the heterogeneity of the patients, the lack of a common classification system, the diverse types of treatment, and the difficulties in comparing data analyses make the choice of endpoints and an objective comparison among these series extremely difficult and inaccurate. The decannulation rate, which was once considered the only element for evaluating the final outcome after BLS treatment, does not allow proper evaluation of the functional state of the larynx and/or the ensuing quality of life; moreover, it makes sense only when referred to preoperatively tracheostomy-dependent patients. As a matter of fact, we obtained a decannulation rate of 63%, nearly overlapping the 66% rate reported by Monnier and coworkers regarding a cohort of 100 patients affected by laryngotracheal stenosis exclusively treated endoscopically [5] and in concordance with the 63% (CI_95%_ 47%–77%) pooled decannulation rate, applying endoscopic treatments for the management of laryngotracheal stenosis, reported in the review by Lewis et al. [19].

In 2007, Nourarei proposed a validated score that could "stage" the condition of the upper airways in terms of airway patency (A), dyspnea (D), voice (V), and swallowing (S) [9, 10]. Based on this score, we identified an improvement in terms of airway status (*p* = 0.008), dyspnea (*p* < 0.0001), and phonation difficulties (*p* < 0.0001) between the pre- and postoperative settings, while there were no significant changes in terms of swallowing (*p* = 0.13), which was already satisfactory before surgery. Moreover, VHI-30 and EAT-10 scores highlight a significant reduction in the negative impact of voice on the quality of life (*p* < 0.0001), while the subjective swallowing function did not change significantly after treatment, in agreement with the ADVS results. Subjective vocal outcome improvement is in agreement with previous literature reporting similar results after the endoscopic treatment of BLS [41, 42], including patients submitted to permanent TLM for bilateral vocal fold palsy alone, showing improvement of physical sub-score of VHI-12 questionnaire [43]. Furthermore, in our cohort the subjective voice improvement, observed also in patients submitted to posterior cordotomy (8 patients), can be explained considering that 7 of them were tracheostomy dependent and 5 were successfully decannulated, this potentially contributing to the improvement of voice-related subjective quality of life scores.

Analyzing potential confounders associated with the quality of life improvement, a higher grade, presence of comorbidities and a higher overall ELS classification score were associated with a worse quality of life result in terms of ADVS score. These results further support the use of the ELS classification for the staging of BLS amenable to endoscopic treatments.

## Conclusion

Even if BLS is a rather rare condition, it should be considered a potentially disabling disease. Analysis of our data confirms that a minimally invasive transoral approach seems to be, in selected fragile patients who are not eligible for an upfront open-neck procedure, a sound alternative to more traditional therapeutic options, even in the presence of moderate stenosis.

The success rates of this treatment policy can guarantee, in selected cases, comparable results in terms of decannulation to those reported in the literature with transoral approaches, improving the quality of life without precluding any further open-neck treatment, if and when necessary.

## Electronic supplementary material

Below is the link to the electronic supplementary material.

Supplementary file1 (PDF 74 kb)
