# Supplementary material for: Transoral management of adult benign laryngeal stenosis
Source: Eur Arch Otorhinolaryngol. 2020 Jul 23;278(1):149–58. doi: 10.1007/s00405-020-06210-5 (PMC7811501; doi:10.1007/s00405-020-06210-5)
Supplement: Supplementary file 1 — Supplementary file1 (PDF 74 kb) [file 405_2020_6210_MOESM1_ESM.pdf]

Supplementary material

Supplementary Figure 1

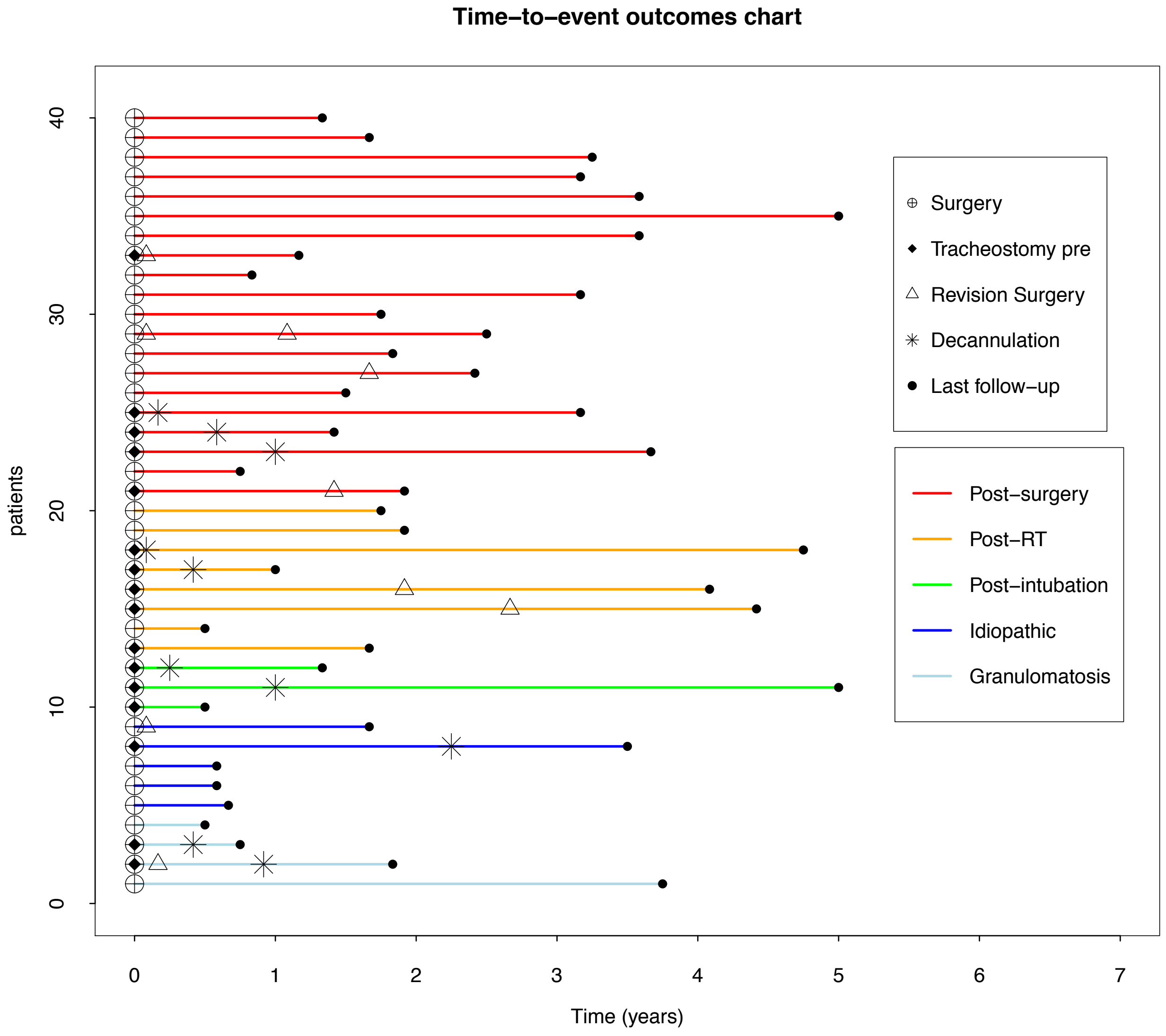

Supplementary Figure 1: Time-to-event outcomes chart showing for each patient the follow-up intervals and timeline of events of interest: tracheostomy at presentation, further surgeries, decannulation time, and last follow-up. Each patient is represented by a horizontal line and the Etiology of stenosis is showed by lines' color, as detailed in the legend.
